# Can cell proliferation of umbilical cord blood cells reflect environmental exposures?

**DOI:** 10.1186/s40064-015-1134-0

**Published:** 2015-07-24

**Authors:** Lena Novack, Esther Manor, Elena Gurevich, Maayan Yitshak-Sade, Daniella Landau, Batia Sarov, Reli Hershkovitz, Doron Dukler, Tali Vodonos, Isabella Karakis

**Affiliations:** Faculty of Health Science, Ben-Gurion University of the Negev, Beersheba, Israel; Genetic Institute, Soroka University Medical Center, Beersheba, Israel; Clinical Research Center, Soroka University Medical Center, Beersheba, Israel; Department of Neonatology, Soroka University Medical Center, Beersheba, Israel; Ultrasound Unit, Department of Obstetrics and Gynecology, Soroka University Medical Center, Beersheba, Israel; Obstetric Emergency Room and Delivery Wards, Soroka University Medical Center, Beersheba, Israel; Environmental Epidemiology Department, Ministry of Health, Jerusalem, Israel; Department of Public Health, Ben-Gurion University of the Negev, P.O.B. 653, Beersheba, Israel

**Keywords:** Cell proliferation, Environmental exposure, Biomarkers, Pregnancy, Umbilical cord

## Abstract

Environmental hazards were shown to have an impact on cell proliferation (CP). We investigated CP of lymphocytes in umbilical cord blood in relation to prenatal environmental exposures in a sample of 346 Arab-Bedouin women giving birth in a local hospital. Information on subjects’ addresses at pregnancy, potential household exposures and demographical status was collected in an interview during hospitalization. This population is usually featured by high rates of neonatal morbidity and multiple environmental exposures, originating from the local industrial park (IP), household hazards and frequent male smoking. A geometric mean CP ratio 2.17 (2.06; 2.29), and was high in women residing in a direction of prevailing winds from the local IP (p value = 0.094) and who gave birth during fall-winter season (p value = 0.024). Women complaining on disturbing exposure to noise had lower CP (p value = 0.015), compared to other women. CP was not indicative of neonatal morbidity. However, our findings suggest that CP of umbilical cord might be modified by environmental exposures. A long-term follow-up of the children is required to assess their developmental outcomes.

## Introduction

Environmental exposures were shown to have an impact on cell life functions, although the research in this direction is scarce. Mice offspring exposed in utero to high levels of particulate matter (PM) showed significantly suppressed splenocyte proliferation (Hong et al. 2013). PM was shown to have an adverse impact on the respiratory system leading to cytotoxic and inflammatory effects, as measured by the cell proliferation MTT test (Orona et al. 2014). Another study (Schaub et al. 2009) showed a decreased lymphocytes proliferation in cord blood taken in mothers exposed to farming during pregnancy. The studies demonstrated a possibility of a modulating effect of environmental factors on fetus cells.

The possible linkage between impaired cell malfunctioning and environment might explain an abundant research connecting environmental pollution with an increased neonatal morbidity worldwide, e.g. birth defects (Padula et al. 2013a, b; Vinikoor-Imler et al. 2013; Ramakrishnan et al. 2013; Agay-Shay et al. 2013a, b; Fung et al. 2013; Nieuwenhuijsen et al. 2013).

The association between cell function and morbidity (specifically, malformations) has been examined in relation to a spectrum of small eye phenotypes in Zebrafish embryos (French et al. 2013). Hypersecretion and oxidative markers of lung injury in mice were linked to environmental exposure to Ozone (O3) (Cho et al. 2013).

This concept, however, is rarely discussed in epidemiological literature.

In the current investigation we aimed to assess the properties of cell proliferation of lymphocytes in umbilical cord blood of pregnant women in relation to hazardous environments in their household and outdoor pollution. We studied the population of Arab-Bedouin pregnant women characterized by excessive rates of major congenital malformations (CM) and other morbidity and mortality, as previously shown in a number of semi-ecological investigations (Bentov et al. 2006; Sarov et al. 2008).

The Arab-Bedouin population is exposed to multiple hazardous factors. It is featured by the lowest socio-economic level (Abu-Saad 1998), a high rate of unemployment, low educational level (The Central Bureau of Statistics 2015), and frequent consanguineous marriages (45%) (Jaber et al. 1994). This population resides in permanent housing, but frequently—in traditional tribal settlements—temporary pre-fabricated shacks or tents. Cooking and heating is often provided by open fire. Smoking is very common among Bedouin men (Abu-Saad 1998). The study area is exposed to frequent dust storms in winter (Ganor 1991; Krasnov et al. 2014; Dayan et al. 2011). A local industrial park (IP) presents an additional potential health hazard, whereas residence in its close proximity was associated with higher rates of major CM, independent of consanguinity in the family (Bentov et al. 2006).

In our study we tested the hypothesis that cell proliferation of lymphocytes in umbilical cord blood of pregnant women is associated with various environmental exposures.

Most of the research performed currently in environmental health is focused on ambient measurements of exposure or on in vitro studies of animals’ cells. (Cho et al. 2013; Pires et al. 2011) However it is the human individual measurement of exposure that is mostly desired for an accurate estimation of the environmental impact. The high cost of biomarkers and their complexity make them unavailable for a large scale study. Umbilical cord is the material which has a potential of a non-invasive testing procedure (always available at delivery) and representing simultaneously maternal and fetal organisms. With this in mind, investigation of the properties of cord blood might enable an accurate estimation of exposure in future environmental research.

## Methods

We enrolled mothers of Arab-Bedouin origin 18 years of age and older and their offspring delivered by singleton birth in Soroka University Medical Center (SUMC) in Beer-Sheva, the only medical center providing tertiary services to the residents in the study area of about 600,000–700,000 residents in southern Israel. We excluded neonates with gestational age under 22 weeks or weight under 500 g, to avoid serious morbidity in the study population most possibly unrelated to the environmental exposures. During June 2012–December 2013, every eligible mother was approached upon her admission to the obstetric emergency room, and was invited to participate in the study, during the day-working hours.

We used the MTT (3-(4,5-dimethylthiazol-2-yl)-2,5-diphenyltetrazolium bromide) *Cell Proliferation Assay* (*Yellow MTT*), which measured a sustainability of cells in umbilical cord. Specifically, the assay tests the proliferation reflecting the mitochondrial function, such as energy metabolism, control of apoptosis and has its own genetic material in addition to nucleus with high ability of generation of reactive oxygen species (ROS) (Byun et al. 2013). The test is considered to be a valuable tool in a wide range of research areas, assessing drug sensitivity, cytotoxicity, response to growth factors, and cell activation (Mosmann 1983). A linear relationship between cell number and absorbance is established for each cell, enabling accurate and straight-forward quantification of changes in proliferation. In the present study the cells were isolated from blood by Ficoll-Histopaque density gradient. As the next step, we used the overall T lymphocyte cells of the umbilical cord blood known to be stimulated by Phytohemagglutinin (PHA). The Optical Density (OD) values of MTT were assessed by the enzyme-linked immunosorbent assay (ELISA). To obtain the cell proliferation ratio, the T cells stimulated by PHA were further divided by an estimate of a natural not-stimulated proliferation of the same cells within the same individual (American Type Culture Collection 2011).

The assay, however, has its limitations since the results might be affected by a physiological state of cells and variance in mitochondrial dehydrogenase activity in different cell types (Cell Proliferation Assay 2015) as well as viral morbidity and smoking (Li et al. 2013; Cheng et al. 2012).

Umbilical cord blood samples were collected at delivery and stored at +2/3°C for up to 48 h before they were processed (Cell Proliferation Assay 2015) for testing.

### Assessment of environmental factors

Information on subjects’ addresses at pregnancy, potential household exposures and demographical status was collected in an interview during hospitalization. The interview included questions regarding subjects’ exact addresses at pregnancy, socio-economic status, family history of malformations, consanguineous marriages, parental exposure to environmental or occupational factors, parental health behavior and health status during the pregnancy and recent medical problems.

An exposure to prevailing winds from the local IP was established in a previous ecological study in the area (Druyan et al. 1986), which remained stable over years. (Cedar Lake Ventures Inc 2014) Clinical information was retrieved from the hospital Admission–Transfer–Discharge (ATD) database.

### Statistical analysis

Continuous data were presented as mean ± standard deviation (SD), median, range and compared between subgroups by t test or Mann–Whitney. Categorical data were presented as proportions and compared using Chi Square or Fisher exact tests. CP ratio—the main outcome variable—was log-transformed and presented as geometric mean and the 95% confidence interval (CI). The CP ratio was compared between two subgroups using a ratio t test, and three or more subgroups using log-normal regression model. For the multivariable analysis of CP ratio readings the log-normal regression model was used, which provided estimates of prevalence ratios (PR) representing the multiplicative difference in CP ratio from the reference category. The choice of the final list of covariates in the reduced and final model was based on the findings of the univariate analysis (p value <0.2), potential confounding of other covariates and statistical significance in the model (p value <0.10). The significance level in the multivariable analysis was set to 10% due to the relatively small sample size.

The written informed consent from subjects participating in the study was received prior to conducting the study. The study has been reviewed and approved by ethics committee (IRB) of the SUMC (#5017).

## Results

Overall 346 women were enrolled in the study. The questionnaires were collected for 286 of the 346 enrolled women (82.7%), whereas 60 women without a questionnaire could not be reached by the interviewers due to early discharge and/or frequently on weekends. These women were 1 year older, resided closer to the IP and their offspring weighted 200 g more than the rest of the sample with questionnaires.

**Fig. 1:**
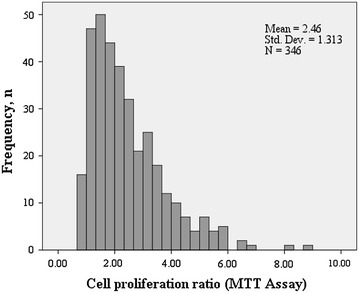
Distribution of cell proliferation values in the study population.

The mean (±sd) of the CP ratio was 2.46 (±1.31), median 2.15, within the range 0.71–8.7 (Fig. 1). The geometric mean of CP ratio was 2.17, with 95% CI 2.06; 2.29. The ratio was further investigated by quartiles, broken by 1.48, 2.15 and 3.15, corresponding to the 25th, 50th and 75th percentiles, and as log-transformed continuous variable.

The women were on average 27.3 ± 5.9 years old and median 26 years. For 30.5% of women it was the first delivery and 22.7%—had a history of more than 6 deliveries. About 10% of the women had a record of lack of prenatal care (LOPC) during pregnancy, usually for older women, and half of them (140/278) reported being married to a relative, usually (in 77.6%) to a first-degree relative. Smoking of a husband was very frequent (94.9%, 261/275).

The demographical factors, including consanguineous marriages, did not impact the CP ratio (Table 1). Some medical conditions, like a recommendation for abortion of the current delivery, was associated with lower CP ratio (GM of CP = 1.43 in women with recommendation vs. 2.20 in women without, p value = 0.016). The history of repeated abortions and self-reported complications in pregnancy had only a borderline tendency towards lower values (p values 0.092 and 0.091, respectively). Lower levels of CP ratio were also detected in umbilical cord of fetuses exposed to parental smoking, however this finding was of borderline significance, as well (0.105). Two mothers with the chronic hypertension diagnosis had high CP ratio (GM = 2.81, compared to GM = 2.16 in the study sample, p value = 0.024).

**Table 1:** Demographic factors and medical history in relation to cell proliferation ratio: 346 Arab-Bedouin women

Patients characteristics	Patients with cell proliferation in				p value	N = 346 (N = 286)	Geometric mean (95% CI)	Prevalence ratio	p value
	Q1: 0.71–1.48	Q2: 1.48–2.15	Q3: 2.15–3.15	Q4: 3.15–8.78					
	N = 86	N = 87	N = 86	N = 87					
Maternal age at delivery, years									
Mean ± SD	26.4 ± 5.0	28.4 ± 6.6	27.1 ± 6.0	27.6 ± 5.9	0.167	<26 163 (47.8)	2.17 (2.02; 2.34)	1.00	
Median	26	27	25	26		≥26 178 (52.2)	2.17 (2.01; 2.36)	1.00^c^	0.988
Parity, % (n)									
1st delivery	29.1% (25)	27.6% (24)	34.9% (30)	28.7% (25)	0.956	104 (30.5)	2.18 (1.99; 2.39)	1.00	
2–5 deliveries	48.8% (42)	47.1% (41)	39.5% (34)	49.4% (43)		160 (46.9)	2.17 (1.99; 2.36)	1.00^c^	0.961
6+ deliveries	20.9% (18)	23.0% (20)	24.4% (21)	20.7% (18)		77 (22.7)	2.17 (1.96; 2.41)	1.00^c^	0.988
Lack of Prenatal Care, % (n)	7.0% (6)	8.0% (7)	10.5% (9)	12.6% (11)	0.586	No: 312 (90.2) Yes: 34 (9.8)	2.15 (2.03; 2.27) 2.41 (2.01; 2.89)	1.00 1.12	0.205
Consanguineous marriage^a,b^, % (n)	46.3% (31)	52.1% (37)	49.3% (34)	53.5% (38)	0.837	No: 138 (49.6) Yes: 140 (50.4)	2.13 (1.96; 2.31) 2.23 (2.08; 2.43)	1.00 1.05	0.434
Relative proximity in consanguineous marriage, % (n)									
1st degree	80.0% (24)	63.6% (21)	76.5% (26)	89.2% (33)	0.141	104 (77.6)	2.30 (2.07; 2.56)	1.00	
2nd degree	20.0% (6)	24.2% (8)	17.6% (6)	5.4% (2)		22 (16.4)	1.90 (1.62; 2.23)	0.83	0.118
Distant relatives	0.0% (0)	12.1% (4)	5.9% (2)	5.4% (2)		8 (6.0)	2.40 (1.84; 3.13)	1.04	0.831
History of repeated abortions	7.0% (6)	6.9% (6)	2.3% (2)	2.3% (2)	0.241	No: 330 (95.4) Yes: 16 (4.6)	2.19 (2.08; 2.31) 1.77 (1.37; 2.28)	1.00 0.81	0.092
Received a recommendation for abortion						No: 278 (97.2) Yes: 16 (2.8)	2.20 (2.08; 2.33) 1.43 (1.03; 1.99)	1.00 0.65	0.016
Chronic disease	11.6% (10)	8.0% (7)	9.3% (8)	13.8% (12)	0.622	No: 337 (97.4) Yes: 9 (2.6)	2.16 (2.05; 2.28) 2.52 (1.62; 3.93)	1.00 1.18	0.359
Diabetes mellitus	8.1% (7)	3.4% (3)	5.8% (5)	9.2% (8)	0.432	No: 338 (97.7) Yes: 8 (2.3)	2.17 (2.05; 2.29) 2.33 (1.46; 3.74)	1.00 1.08	0.68
Chronic hypertension	0.0% (0)	0.0% (0)	0.0% (0)	2.3% (2)	0.112	No: 344 (99.4) Yes: 2(0.6)	2.16 (2.05; 2.28) 4.81 (4.00; 5.79)	1.00 2.23	0.024
Self-reported complications in pregnancy^a^, % (n)	1.4% (1)	2.7% (2)	0.0% (0)	0.0% (0)	0.312	No: 283 (99.0) Yes: 3 (1.0)	2.19 (2.06; 2.32) 1.34 (0.58; 3.07)	1.00 0.61	0.091
Paternal smoking^a^, % (n)	98.4% (63)	95.8% (69)	92.8% (64)	92.9% (65)	0.384	No: 14 (5.1) Yes: 261 (94.9)	2.70 (2.03; 3.60) 2.16 (2.04; 2.30)	1.00 0.8	0.105

^a^Information on consanguineous marriages was available for 80.3% of the sample, complications in pregnancy: for 82.7% of the sample and paternal smoking: for 79.5%.

^b^Consanguineous marriages differ by their degree of proximity between the married relatives. The first degree is assigned to the parents who are also the first cousins, the second degree: to the second cousins and the category of the “distant relatives” is assigned to more distant relationships in the family.

^c^Value “1.00” indicates a very small effect on CP ratio approximating “1”.

About 25% of women reported living in a shack or tent (69/286) (Table 2). Residing in a multi-story building yielded lower levels of CP (GM = 1.99, p value = 0.052) compared to others. Above 89% of the study participants complained on exposure to dust and 55.6% reported car usually parked next to their house as a disturbing factor, which was more prevalent in women with higher cell proliferation (p value = 0.052), however this association was not linear (p value = 0.297). Usage of open fire was expectantly high for heating (10.1%) and cooking (74.5%), but showed no impact on cell proliferation.

**Table 2:** Environmental Factors in relation to cell proliferation ratio: 346 Bedouin Women

Environmental factors^a^	Patients with proliferation in				p value	N = 346 (N = 286)	Geometric mean (95% CI)	Prevalence ratio	p value
	Q1: 0.71–1.48	Q2: 1.48–2.15	Q3: 2.15–3.15	Q4: 3.15–8.78					
	N = 86	N = 87	N = 86	N = 87					
	(N = 69)^a^	(N = 74)^a^	(N = 70)^a^	(N = 73)^a^					
Type of a house, % (n)									
Shack/tent	21.7% (15)	21.6% (16)	28.6% (20)	24.7% (18)	0.147	69 (24.6)	2.18 (1.95; 2.44)	1.00	
One-story building	42.0% (29)	47.3% (35)	41.4% (29)	54.8% (40)		133 (47.5)	2.30 (2.10; 2.51)	0.96	0.534
Multi-story building	33.3% (23)	28.4% (21)	28.6% (20)	19.2% (14)		78 (27.9)	1.99 (1.78; 2.23)	0.87	0.052
Environmental factors reported as disturbing, % (n)									
Dust	87.0% (60)	91.9% (68)	87.1% (61)	91.8% (67)	0.627	No: 30 (10.5) Yes: 256 (89.5)	2.09 (1.76; 2.48) 2.18 (2.05; 2.32)	1.00 1.05	0.634
Mosquitoes	91.3% (63)	86.5% (64)	92.9% (65)	87.7% (64)	0.561	No: 30 (10.5) Yes: 256 (89.5)	2.38 (1.96; 2.88) 2.15 (2.02; 2.29)	1.00 0.91	0.303
Noise	10.1% (7)	8.1% (6)	2.9% (2)	4.1% (3)	0.247	No: 268 (93.7) Yes: 18 (6.3)	2.20 (2.08; 2.34) 1.78 (1.42; 2.24)	1.00 0.81	0.082
Waste	4.3% (3)	10.8% (8)	2.9% (2)	9.6% (7)	0.172	No: 266 (93.0) Yes: 20 (7.0)	2.16 (2.04; 2.30) 2.34 (1.82; 3.00)	1.00 1.08	0.501
Transport (family cars)	53.6% (37)	44.6% (33)	57.1% (40)	67.1% (49)	0.052	No: 127 (44.4) Yes: 159 (55.6)	2.10 (1.94; 2.28) 2.24 (2.06; 2.43)	1.00 1.06	0.297
Type of heating, % (n)									
Electric/AC/central heating	34.8% (24)	48.6% (36)	37.1% (26)	34.2% (25)	0.245	No: 175 (61.2) Yes: 111 (38.8)	2.19 (2.03; 2.37) 2.14 (1.96; 2.34)	1.00 0.98	0.703
Stove with chimney	10.1% (7)	9.5% (7)	10.0% (7)	6.8% (5)		No: 260 (90.9) Yes: 26 (9.1)	2.19 (2.05; 2.33) 2.06 (1.76; 2.41)	1.00 0.94	0.571
Stove without chimney	43.5% (30)	25.7% (19)	38.6% (27)	46.6% (34)		No: 176 (61.5) Yes: 110 (38.5)	2.15 (2.01; 2.30) 2.20 (1.99; 2.47)	1.00 1.03	0.581
Open fire	7.2% (5)	10.8% (8)	10.0% (7)	12.3% (9)		No: 257 (89.9) Yes: 29 (10.1)	2.16 (2.03; 2.29) 2.14 (1.95; 2.80)	1.00 1.08	0.422
Cooking on open fire, % (n)	76.8% (53)	67.6% (50)	74.3% (52)	79.5% (58)	0.39	No: 73 (25.5) Yes: 213 (74.5)	2.11 (1.89; 2.35) 2.20 (2.05; 2.36)	1.00 1.04	0.525
Usage of pesticides, % (n)	91.3% (63)	86.5% (64)	90.0% (63)	86.3% (63)	0.723	No: 33 (11.5) Yes: 253 (88.5)	2.42 (2.03; 2.89) 2.14 (2.02; 2.28)	1.00 0.88	0.186
Using water containers, % (n)	91.3% (63)	86.5% (64)	92.9% (65)	93.2% (68)	0.47	No: 26 (9.1) Yes: 260 (90.9)	2.18 (1.79; 2.66) 2.17 (2.04; 2.31)	1.00 1.00	0.974
Type of water container, % (n)									
Water tank	26.1% (18)	33.8% (25)	28.6% (20)	19.2% (14)	0.109	No: 209 (73.1) Yes: 77 (26.9)	2.22 (2.06; 2.38) 2.07 (1.87; 2.28)	1.00 0.93	0.927
Small containers	60.9% (42)	47.3% (35)	57.1% (40)	63.0% (46)		No: 123 (43.0) Yes: 163 (57.0)	2.15 (1.98; 2.33) 2.19 (2.02; 2.38)	1.00 1.02	0.728
Barrel	4.3% (3)	5.4% (4)	7.1% (5)	11.0% (8)		No: 266 (93.0) Yes: 20 (7.0)	2.16 (2.03; 2.29) 2.45 (1.98; 3.02)	1.00 1.14	0.27
Resides in direction of prevailing wind from IP, % (n)^b^	14.0% (12)	14.9% (13)	23.3% (20)	28.7% (25)	0.046	No: 276 (79.8) Yes: 70 (20.2)	2.11 (1.99; 2.24) 2.44 (2.16; 2.74)	1.00 1.16	0.03
Resides within 10 km from IP^a^, % (n)^b^	7.2% (6)	5.8% (5)	9.5% (8)	8.3% (7)	0.827	No: 311 (92.3) Yes: 26 (7.7)	2.16 (2.05; 2.29) 2.24 (1.85; 2.71)	1.00 1.03	0.741
Delivery in fall or winter, % (n)^b,c^	68.6 (59)	64.4 (56)	75.6 (65)	82.5 (71)	0.056	No: 95 (24.5) Yes: 251 (72.5)	2.00 (1.83; 2.19) 2.24 (2.10; 2.39)	1.00 1.12	0.06

^a^Number of available questionnaires.

^b^Data derived from a database for all subjects.

^c^Fall and winter period was defined as months from September through February.

Women with CP ratio above median were more frequently residing in the direction of prevailing wind from the local IP compared to others (26.0 vs. 14.4%, p value = 0.008, p value for trend = 0.008), however the proximity to the IP made no difference on CP values. Delivery in fall or winter (September thru February), was more frequent for higher levels of proliferation (above median, 78.6%) compared to lower levels of this biomarker, featured by less deliveries in a colder season (66.5%) (p value = 0.011). Of note, the cold season is the one characterized by more frequent winds from the IP.

Noteworthy, complaints on noise featured women with lower CP ratio (GM = 1.78 vs. GM = 2.20 in the rest of the sample), however this finding was only borderline significant (p value = 0.082).

Multivariable analysis indicated an independent positive association of CP with the residence in the direction of the prevalent wind from the local IP (PR = 1.17, p value = 0.075), delivery between September–February (PR = 1.15, p value = 0.028), and a negative association with complaints on noise (PR = 0.81, p value = 0.075). All the parameters above were adjusted to each other and the history of repeated abortions, shown to be related to lower levels of CP (Table 3).

**Table 3:** An effect of environmental factors on the cell proliferation, based on log-normal multivariate model

Environmental factor	Ratio^a^ [95% confidence interval (CI)]	p value
Complaint on noise (n = 18) vs. subjects not complaining on this factor (n = 268)	0.82 (0.65; 1.03)	0.094
Living in a direction of a prevalent wind from local IP (n = 70) vs others (n = 216)	1.18 (1.03; 1.35)	0.015
Delivery in fall-winter^b^ (n = 201) vs. deliveries in spring-summer (n = 85)	1.16 (1.02; 1.31)	0.024

^a^Ratios represent the multiplicative difference in cell proliferation ratio from the reference category.

^b^Fall and winter period was defined as months from September through February.

Overall, 6.6% (23/346) of enrolled newborns were diagnosed with congenital malformations. Malformations of any type were not found related to the biomarker. Mothers of newborns with malformations had experienced more abortions in the past, compared to mothers of healthy newborns (13.6 vs. 4.6%, p value = 0.009) (data not shown).

## Discussion

In this investigation we examined an effect of environmental exposures on a biomarker of an impaired or altered cell functions in humans, specifically, cell proliferation.

Readings of CP were found reflective of environmental exposures. The direction of CP ratio change towards higher values or lower values depended on the type of the exposure and is quite intriguing. For instance, in the presence of most of the external environmental factors the CP ratio values were likely to be higher, e.g. residence downwind from the local IP or cold seasons. This finding was supported by a study in mice showing increased inflammatory processes in the animals’ cells as a result of exposure to Ozone (Cho et al. 2013). However, the report on cytotoxicity in human alveolar epithelial cells following exposure to particles was not consistent as well, showing once reduced, increased or unchanged cell viability (Orona et al. 2014). The exposure to noise in our study had an opposite tendency of decreasing the CP ratio. This finding is reinforced by the report by Jáuregui-Huerta et al. (2011) on exposure of rats to environmental noise associated with decreased cell proliferation in the hippocampal formation.

In some instances, associations of environmental factors and CP in a univariate exploratory analysis did not follow a linear dose–response function, but rather a non-monotonic dose response curves, e.g. exposure to family cars parked closely to the women’s house was correlated with both, low and high CP ratio. This type of association, resembling a U-shape, is often described in epidemiology, as well (Vandenberg 2013).

Lower CP levels, indicating low cellular metabolic activity, were more likely to be reported in mothers with adverse obstetrical history, i.e. repeated abortions and self-reported complications. Romanelli et al. (2009) team also expected preeclampsia to be the negative regulator of cell proliferation.

To conclude our main findings, we can hypothesize that the CP ratio is differently affected by external and internal factors, whereas higher values probably indicated environmental exposures and lower values—maternal and possibly neonatal morbidity. The example of exposure to noise contradicts this logic, however. Nevertheless, the mechanism could not be affirmed with the small sample in the current investigation, but should be tested in future research.

The absence of statistically significant association of CP ratio with the neonatal health outcomes, specifically malformations could be explained by a relatively small sample. Furthermore, if lower CP is indeed related to the history of repeated abortions or other complications (as indicated in the study), the fetuses with serious impairments might have been aborted prior to their enrollment, which in turn would limit the CP range to relatively higher values and make the differences indistinguishable. Furthermore, the CP values at birth might be associated with the newborn morbidity as revealed further in life, which requires a longer follow-up.

Of note, we registered a very high percent of consanguineous marriages in the study population, especially for the 1st degree relatives. This estimate was expected in the current population and is supported by reports by Jaber et al. (1994).

The population-based setting of the study, in which the participants were not pre-selected by the choice of a hospital and laboratory personnel; using a questionnaire with detailed description of the women’s household and individual factors; and the biomarker representing both—maternal and neonatal organisms—all these add to the validity of our findings.

The study has its limitations.

- Timing of the cell proliferation measurement taken only at delivery and not measured during pregnancy imposes a limitation, especially for investigation of malformations, whereas an early pregnancy period would be the most relevant. However, if women do not change their residence throughout their pregnancy (as in our study population), their cell proliferation values might reasonably well reflect the environmental exposures we meant to capture in the analysis, after a necessary adjustment to seasonality.
- We did not interview all the women enrolled in the study and, therefore, missed information on household exposures for 17% of the study sample. Based on the hospital charts, these women were more frequently residing closer to the IP than women who were interviewed. Therefore, there is a possibility of a selection bias, which might have weakened associations obtained in the analysis.
- It is essential to stress that the MTT assay that reflects viable cell metabolism, providing only a general indication of a cell function (proliferation) per se. Therefore, in a future research using this assay, a cell genetic testing of micronucleus or DNA damage, should be considered for validation of the results.
- We did not find an association of CP ratio with residing within 10 km from the IP, however, testing for even higher proximity, e.g. 5 km, was not feasible due to the small concentrations of the population within this radius from the IP. This limitation might have created a certain misclassification of exposure leading to the non-significant result.
- In addition, severe cases of preterm deliveries (<22 weeks) and low-birth weight (<500 g) were not included in the current investigation, which might have resulted in a selection bias towards a healthier population. While most of extreme cases of morbidity were not related to environment, our exclusion criteria might have also excluded cases with extreme hazardous environment and hence, decreased the magnitude of associations of CP and environmental factors.

## Conclusions

The study suggests that the cell proliferation is independently associated with some environmental exposures. A long-term follow-up investigation of the children is required to assess their cognitive development. Women presenting with low or high levels of CP ratio should be followed-up.
